# An Image Registration Method for Multisource High-Resolution Remote Sensing Images for Earthquake Disaster Assessment

**DOI:** 10.3390/s20082286

**Published:** 2020-04-17

**Authors:** Xin Zhao, Hui Li, Ping Wang, Linhai Jing

**Affiliations:** 1 College of Geomatics, Shandong University of Science and Technology, Qingdao 266590, China; zhaox_sd@163.com (X.Z.); pingwsd@163.com (P.W.); 2 Key Laboratory of Digital Earth Science, Aerospace Information Research Institute, Chinese Academy of Sciences, Beijing 100094, China; jinglh@radi.ac.cn; 3 Hainan Key Laboratory of Earth Observation, Sanya 572029, China

**Keywords:** image registration, multisource high-resolution remote sensing image, earthquake damage assessment, Shi_Tomasi corner detection algorithm, SIFT

## Abstract

For earthquake disaster assessment using remote sensing (RS), multisource image registration is an important step. However, severe earthquakes will increase the deformation between the remote sensing images acquired before and after the earthquakes on different platforms. Traditional image registration methods can hardly meet the requirements of accuracy and efficiency of image registration of post-earthquake RS images used for disaster assessment. Therefore, an improved image registration method was proposed for the registration of multisource high-resolution remote sensing images. The proposed method used the combination of the Shi_Tomasi corner detection algorithm and scale-invariant feature transform (SIFT) to detect tie points from image patches obtained by an image partition strategy considering geographic information constraints. Then, the random sample consensus (RANSAC) and greedy algorithms were employed to remove outliers and redundant matched tie points. Additionally, a pre-earthquake RS image database was constructed using pre-earthquake high-resolution RS images and used as the references for image registration. The performance of the proposed method was evaluated using three image pairs covering regions affected by severe earthquakes. It was shown that the proposed method provided higher accuracy, less running time, and more tie points with a more even distribution than the classic SIFT method and the SIFT method using the same image partitioning strategy.

## 1. Introduction

Disasters caused by severe earthquakes, such as collapsed buildings, road damage, dammed lakes, and secondary geological disasters, pose a threat to people’s lives and property safety worldwide. In recent years, major earthquakes that occurred in China, such as the Wenchuan earthquake on 12 May 2018, and the Yushu earthquake on 14 April 2010, attracted considerable attention from the government and society. Obtaining exact disaster information immediately after severe earthquakes is important for disaster rescue and relief and can effectively reduce the damage caused by severe earthquakes [1,2]. With the advantages of wide-ranging, multiscale, dynamic comprehensive observation and being free from ground conditions, remote sensing (RS) technology for earth observation provides a rapid, safe, and economical method for earthquake disaster assessment and emergency rescue [3]. Since the occurrence of the Wenchuan earthquake on May 12, 2018, satellite and airborne RS technologies have played an important role in the rapid acquisition and dynamic monitoring of earthquake disaster information and post-earthquake reconstruction. 

The data processing for extracting earthquake disaster information from RS images includes image preprocessing, disaster information extraction, and disaster assessment [4]. The accuracy and efficiency of image preprocessing, mainly referring to geometric rectification of RS images and the coregistration of multisource RS images, are crucial for the accuracy and efficiency of disaster information extraction. In order to obtain disaster information quickly and support disaster responses, it is particularly important to have geometrically accurate RS images available shortly after the acquirement of post-earthquake RS images. Using the images to extract the disaster information, including the location, distribution, and extension, is very crucial for disaster relief and rescue. For instance, in transportation planning processes after an earthquake, a fast and accurate assessment of the destruction degree of traffic facilities (such as roads and bridges) is essential to have an updated configuration of infrastructures to facilitate disaster relief work [5]. The accuracy of coregistration of the post-earthquake and pre-earthquake RS images is particularly important when change detection methods are used for disaster information extraction.

With the development of RS technology, different satellite sensors can provide multispectral, multitemporal, and multiplatform RS images. Additionally, the spectral resolution of RS images is also continually improving, and image registration research has gradually shifted from medium-low resolution to high resolution [6]. The registration of high-resolution RS images is significantly more challenging than that of medium-low-resolution RS images, since high-resolution RS images contain more intricate details and texture information [7]. Therefore, the coregistration of multisource high-resolution RS images has been a research focus in RS image processing.

Image registration is the process of the geometric registration of two or more images covering the same area and acquired at different times, from different viewpoints, or by different sensors [8]. The majority of the automatic registration methods for RS images can be classified into two categories, including gray-based (or intensity-based) methods and feature-based methods [9,10]. To extract tie points from the two images, the gray-based methods establish a similarity measure between the two images through the mutual information (MI) method [11,12] or normalized cross-correlation method (NCC) [13]. As gray values of pixels within the neighborhood of each pixel need to be considered, this kind of method suffers from a large number of calculations and relatively low efficiency. The feature-based methods locally extract points, lines, and regional features for image registration [14]. Due to the advantages of easy acquisition, short running time, and high robustness, point features are widely used in image registration. Currently, many feature-based methods have been successfully used for RS image registration, such as the Moravec corner detection algorithm [15], the Harris corner detection algorithm [16], the Shi_Tomasi corner detection algorithm [17], the scale-invariant feature transform (SIFT) [18], speeded up robust features (SURF) [19], and the features from accelerated segment test (FAST) [20].

The performances of several different algorithms, including SIFT, shape context [21], steerable filters [22], principal components analysis-SIFT (PCA-SIFT) [23], differential invariants [24], and other methods, were compared by Mikolajczyk and Schmid [25]. The experimental results show that the SIFT algorithm yielded the best performance. However, it was apt to be affected by image noise and texture changes and was computationally intensive and time-consuming during feature point extraction [26]. To solve these problems, several improved versions of the SIFT algorithm were proposed. For instance, the independent component analysis-SIFT (ICA-SIFT) algorithm, which uses independent component analysis to remove redundant information in the SIFT feature vectors, was proposed [27]. It improved the efficiency but decreased the accuracy. The affine-SIFT (ASIFT) algorithm proposed by Yu and Morel [28] achieved complete affine invariance by simulating longitude and latitude, but its efficiency was much lower than that of the SIFT algorithm. The uniform robust SIFT (UR-SIFT) algorithm [29] is suitable for multisource optical RS images with different illumination conditions, rotations, and five-fold scales. However, the crossmatching approach employed by the UR-SIFT algorithm eliminated a number of correctly matched tie points, which reduced the accuracy. Inspired by the SIFT algorithm, an enhanced feature matching method by combining the position, scale, and orientation of each keypoint, which is called PSO-SIFT, was proposed [30]. The PSO-SIFT method effectively increased the number of correctly matched tie points, but it also increased the running time. However, for images with complex terrain and surface deformations caused by disasters, these algorithms can hardly meet the requirements of accuracy and efficiency simultaneously. Through the improvement of the corner response function of Harris, the Shi_Tomasi corner detection algorithm is not susceptible to image rotations, lighting conditions, angle changes, and noise. Additionally, it avoids the phenomenon of feature point clustering, which makes the distribution of extracted feature points more even [31]. Additionally, the Shi_Tomasi algorithm needed less running time than the SIFT algorithm and thus effectively improved the efficiency of feature extraction.

Due to surface deformations caused by severe earthquakes, post-earthquake RS images usually show surface damage such as building collapse, ground displacements, road damage, and landslides, making it more challenging for the coregistration of pre- and post-earthquake RS images. Many algorithms work well for RS image registration, but when used for the registration of pre-earthquake and post-earthquake RS images, especially for mountain areas with complex terrain, two problems arise, including the lack of tie points and the uneven distribution of matched tie points. To solve these two problems, a fast automatic registration method for multisource high-resolution RS image registration in earthquake damage assessment was proposed in this study. In this method, the Shi_Tomasi and SIFT algorithms were combined to obtain tie points using an image partition matching strategy considering geographic information. The proposed method was compared with the classic SIFT method and the SIFT method using the same image partitioning strategy using three pairs of post- and pre-earthquake images that have complex terrain and significant differences in color tone. 

The rest of this paper is organized as follows. The proposed method is introduced in Section 2, and the experiments are demonstrated in Section 3. The discussions are provided in Section 4, and the conclusions are presented in Section 5.

## 2. Methodology

**Figure 1:**
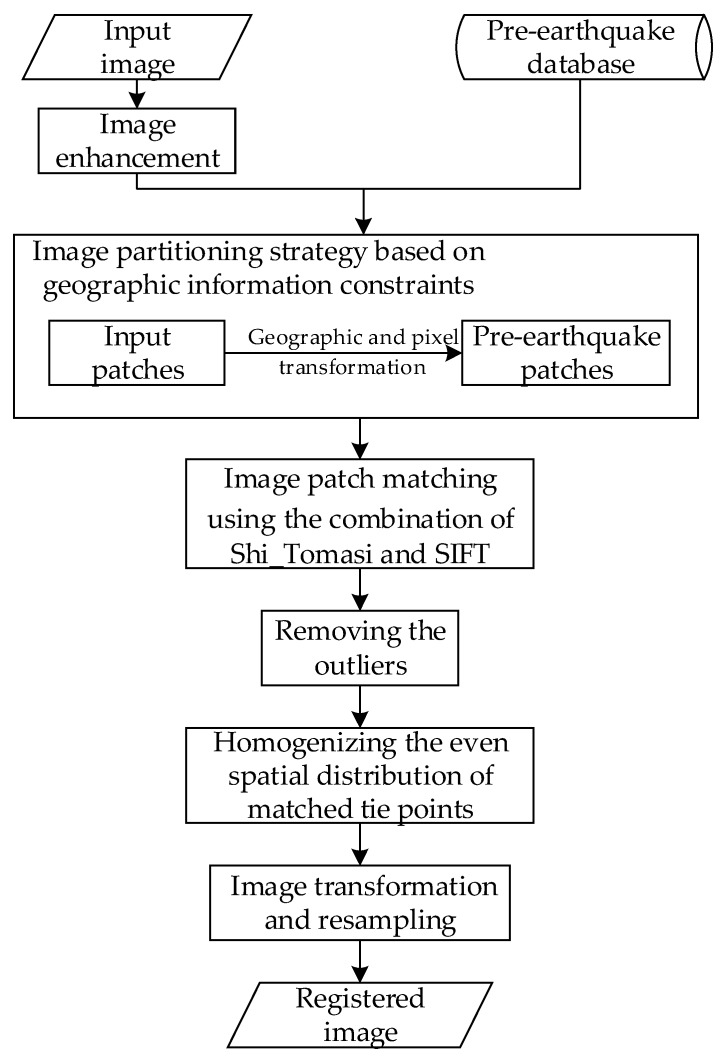
Flowchart of the proposed method.

The proposed method employs an improved SIFT algorithm using the Shi_Tomasi approach to detect feature points on enhanced images and a partition matching strategy considering geographic information to search for tie points. Seven steps are involved in the method (Figure 1), as follows:

(1) Constructing a pre-earthquake image database;
(2) Image enhancement;
(3) Image partitioning strategy based on geographic information constraints;
(4) Image patch matching using the combination of Shi_Tomasi and SIFT;
(5) Removing the outliers;
(6) Homogenizing the even spatial distribution of matched tie points;
(7) Image transformation and resampling.

### 2.1. Constructing a Pre-Earthquake Satellite Image Database

To improve the efficiency and the degree of automation of image processing, a pre-earthquake image database can be first constructed using high-resolution optical remote sensing images covering the study area. The construction of the database mainly consists of three steps. First, radiometric calibration, atmospheric correction, and projection coordinate conversion are used to preprocess the pre-earthquake high-resolution satellite images. Then, the preprocessed images are geometrically corrected according to ground control points (GCPs) if GCPs are available. If no GCPs are available in the case of emergency, high-resolution pre-earthquake RS images with high quality and strict orthorectification can be used without geometric correction. Finally, the identity number (ID) and the coordinates of the corresponding high-resolution satellite image are recorded in a coordinate file, as shown in Table 1. The pre-earthquake RS images and the corresponding coordinate files were stored in the same directory to construct the pre-earthquake satellite image database.

### 2.2. Image Enhancement

Usually, image denoising is employed to reduce image noise before image matching. However, the denoised images may suffer from relatively low image contrast when the image has only a single channel or the color change is not obvious [32]. This makes it difficult to extract feature points. To enhance the details of the denoised images and increase the number of extracted feature points, the proposed method adopted a 2% linear stretch method to enhance both the input and the reference images.

The 2% linear stretch method is usually performed by defining a transfer function in the following form:

(1) $$g(x,y)=\{\begin{array}{cc} c, & 0\leq f(x,y)<a\\ \frac{d-c}{b-a}\left[f(x,y)-a\right]+c, & a\leq f(x,y)<b\\ d, & b<f(x,y) \end{array},$$

where *g* (*x, y*) is the value of pixel (*x, y*) after image stretching, *f* (*x, y*) is the original value of pixel (*x, y*), and *a* and *b* are the values at 2% and 98%, respectively, of the cumulative frequency of the original image. *c* and *d* are the minimum and the maximum of the stretched image, respectively. In this work, *c* = 0, and *d* = 255.

**Figure 2:**
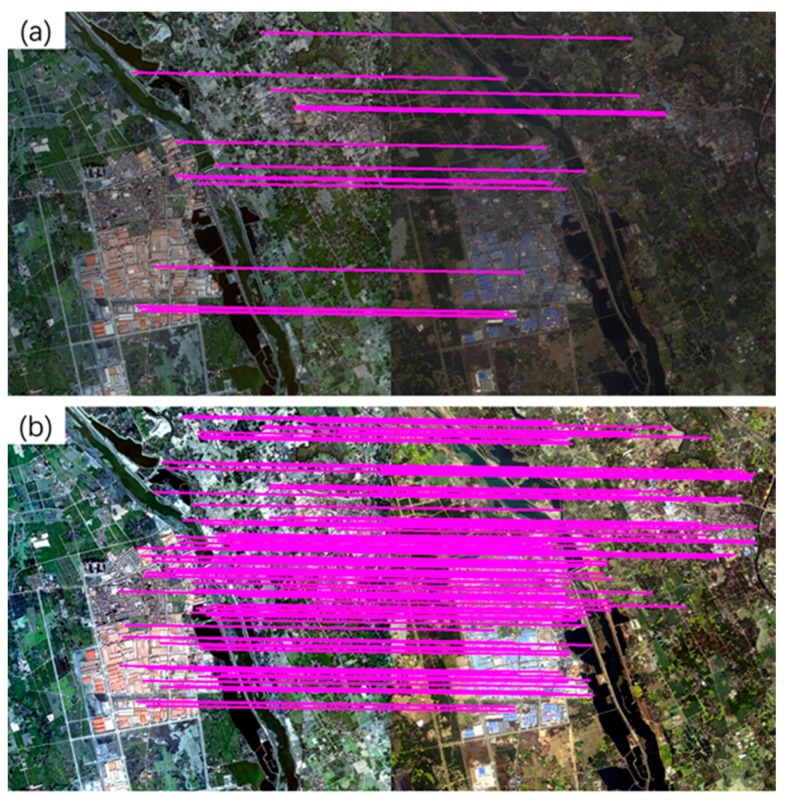
Tie points obtained using the original multispectral image (**a**) and the enhanced image obtained using the 2% linear stretch method (**b**).

To test the advantage of the 2% linear stretch method, an experiment was performed using the GaoFen-1 (GF-1) multispectral image (with an 8-m resolution) and the SIFT algorithm. The original image yielded 16 matched tie points, as shown in Figure 2a. In contrast, the enhanced image obtained 119 matched tie points (Figure 2b). The experimental results show that applying the 2% linear stretching method on the images can facilitate the extraction of feature points and increase the number of matched tie points.

### 2.3. Image Partitioning Strategy Based on Geographic Information Constraints

For large high-resolution remote sensing images, the extraction and matching of feature points usually have high computational complexity, which requires considerable memory and is time-consuming. In addition, it yields a low accuracy of registration when enough feature points are extracted from only some of the regions of the image. To solve this problem, an image partitioning strategy based on geographic information constraints was adopted to divide remote sensing images into several image patches. For each image patch, the corresponding reference image can be obtained according to the projected coordinates of the image patch. The extraction and matching of feature points were applied to each image patch pair.

**Figure 3:**
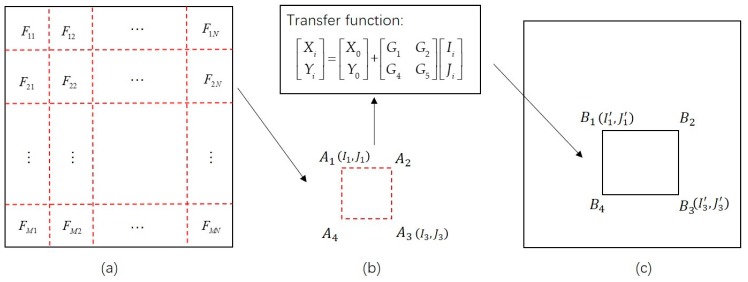
Schematic of the image partitioning strategy based on a geographical information constraint: (**a**) an input image and the image partition of the input image; (**b**) an image patch *F_2N_* (with corners *A*_1_, *A*_2_, *A*_3_, and *A*_4_) of the input image; (**c**) the corresponding reference image patch (with corners *B*_1_, *B*_2_, *B*_3_, and *B*_4_) in a pre-earthquake image covering the input image patch.

With respect to the geometric information of RS images, the mapping relationship [33] between the image coordinates and geographic coordinates can be expressed using Equation (2).

(2) $$\left[\begin{array}{c} X_{i}\\ Y_{i} \end{array}\right]=\left[\begin{array}{c} X_{0}\\ Y_{0} \end{array}\right]+\left[\begin{array}{cc} G_{1} & G_{2}\\ G_{4} & G_{5} \end{array}\right]\left[\begin{array}{c} I_{i}\\ J_{i} \end{array}\right],$$

where (*I_i_, J_i_*) are the image coordinates of the *i*th pixel, and (*X_i_, Y_i_*) are the corresponding projected coordinates of (*I_i_, J_i_*); (*X_0_, Y_0_*) are the projected coordinates of the top left corner in the original whole scene input image, and *G*_1_, *G*_2_, *G*_4_, and *G*_5_, are the parameters of the transformation model. Figure 3 shows a schematic of the image partitioning strategy. The image partition of the input RS image and the corresponding reference image can be conducted using the following steps:

First, the input image is divided into *n×n* patches, each of which has a size of *M×N* pixels. Each patch is numbered, and the coordinates of the four corners of the patch are recorded. For example, the corners of patch *F_2N_* are recorded as *A*_1_, *A*_2_, *A*_3_, and *A*_4_. The image coordinates of corners *A*_1_ and *A*_3_ are then calculated and recorded as *(I*_1_*, J*_1_*)* and *(I*_3_*, J*_3_*)*, respectively.

Second, the image coordinates are transformed using Equation (2) into the projected coordinates according to the geometric information of the input image. Specifically, (*X_0_, Y_0_*) are the projected coordinates of the top left corner in the original whole scene input image; (*I_i_, J_i_*), *i* = 1 or *3* are the image coordinates of corners *A*_1_ and *A*_3_ of the image patch *F_2__N_*; (*X_i_, Y_i_*), *i* = 1 or 3 are the projected coordinates of *A*_1_ and *A*_3_; *G*_1_ is the pixel size of the input image; *G*_5_ is the negative value of pixel size of the input image; and the values of *G*_2_ and *G*_4_ are equal to 0.

Third, according to the projected coordinates *(X_i_, Y_i_)* of an input image patch, the pre-earthquake satellite image covering this patch is found through the pre-earthquake high-resolution image database. If there is more than one image covering the patch, the reference image is the one whose spatial resolution is closest to the input image. Additionally, the extension of the corresponding reference patch can be determined according to *(X_i_, Y_i_)*. The image coordinates (*(I*_1_*^’^, J*_1_*^’^)*, *(I*_3_*^’^, J*_3_*^’^)*), which correspond to the two corners (*B*_1_ and *B*_3_) of the reference image patch shown in Figure 3c, are calculated using Equation (2).

Finally, the image coordinates of the other two corners (*B*_2_ and *B*_4_) of the reference image patch can be obtained according to those of corners *B*_1_ and *B*_3_. The image coordinates of the four corners *B*_1_, *B*_2_,* B*_3_, and *B*_4_ are then used to obtain the corresponding reference image patch from the pre-earthquake satellite image.

### 2.4. Partition Matching Using Shi_Tomasi and SIFT

The Shi_Tomasi and SIFT algorithms were combined to extract feature points from each of the image patch pairs obtained according to the introduction in Section 2.3. The Shi_Tomasi algorithm was used to detect feature points from the image pairs. The SIFT descriptor, which is invariant of scale and rotation, was used to describe the features of detected feature points. The Best Bin First (BBF) algorithm [34] was employed to match the feature points.

#### 2.4.1. Feature Detection Using the Shi_Tomasi Algorithm

The Shi_Tomasi corner detection algorithm is an improved version of the Harris corner detector. Considering a local window in the image, Harris corner points are detected based on the determination of the average changes in image intensity that result from shifting the local window by a small amount in various directions. 

Denoting the image intensity of the image as *I*, the intensity difference (*E*) produced by a shift (*u, v*) of the local window is provided by Equation (3):

(3) $$E(u,v)=\sum_{x,y}w(x,y){\left[I(x+u,y+v)-I(x,y)\right]}^{2},$$

where *w* (*x, y*) is a window function at position (*x, y*).

To search for the windows that produce a large *E* (*u, v*), *I* (*x + u, y + v*) can be expanded using the Taylor series:

(4) $$I(x+u,y+v)=I(x,y)+I_{x}u+I_{y}v+ο(u^{2},v^{2}),$$

where *I_x_* and *I_y_* are image derivatives in the *x* and *y* directions, respectively. Combining Equations (3) and (4), we obtain Equation (5) as:

(5) $$\begin{array}{ll} E(u,v) & =\sum_{(x,y)\in o}\left[\begin{array}{cc} u & v \end{array}\right]w(x,y)\left[\begin{array}{cc} I_{x}^{2} & I_{x}I_{y}\\ I_{x}I_{y} & I_{y}^{2} \end{array}\right]\\ & ≅\left[\begin{array}{cc} u & v \end{array}\right]M\left[\begin{array}{c} u\\ v \end{array}\right] \end{array},$$

where *M* is defined using Equation (6),

(6) $$M=w(x,y)\left[\begin{array}{cc} I_{x}^{2} & I_{x}I_{y}\\ I_{x}I_{y} & I_{y}^{2} \end{array}\right],$$

Finally, a score *R* is calculated using Equation (7) and used to justify whether the window contains a corner:

(7) $$R=detM-k{\left(traceM\right)}^{2},$$

where *detM =* *λ*_1_*λ*_2_; *trace(M) = λ*_1_ + *λ*_2_; and *λ*_1_ and *λ*_2_ are the eigenvalues of *M*. *K* is an empirical value, and the general value of *K* is 0.04 [35]. If the value of *R* is greater than a threshold value (*T*), the window in which *R* is treated as a corner point is a good tracking point.

Different from the Harris algorithm, the score *R* for the Shi_Tomasi algorithm is calculated using Equation (8).

(8) $$R=min(\lambda_{1},\lambda_{2}),$$

**Figure 4:**
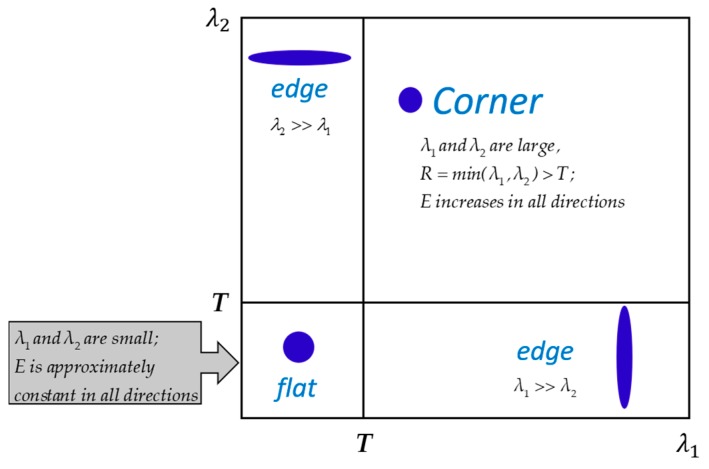
Schematic of the Shi_Tomasi algorithm.

If *R* is greater than *T*, the point can be marked as a Shi_Tomasi corner. The schematic of the Shi_Tomasi algorithm is shown in Figure 4. In this work, the maximum number of corner points (*N_max_*) for each image patch should be set in advance, usually to 1500.

#### 2.4.2. Feature Description Using the SIFT Descriptor

The SIFT descriptor was employed for feature description of the feature points detected in this work. The SIFT descriptor proposed by Lowe in 2004 is a local feature descriptor based on the gradient distribution in the detected regions. By assigning one or more consistent orientations to each feature point based on the gradient directions of a local image, the feature descriptor of the feature point can be represented relative to the orientations and therefore achieve invariance to image rotation. For each feature point (x, y) of the scale image *L*, the gradient magnitude *m (x, y)* and orientation *θ (x, y)* can be calculated using Equations (9) and (10), respectively.

(9) $$m(x,y)={\left[{\left(L(x+1,y)-L(x-1,y)\right)}^{2}+{\left(L(x,y+1)-L(x,y-1)\right)}^{2}\right]}^{\frac{1}{2}},$$

(10) $$\theta (x,y)=arctan(\frac{L(x,y+1)-L(x,y-1)}{L(x+1,y)-L(x-1,y)}),$$

**Figure 5:**
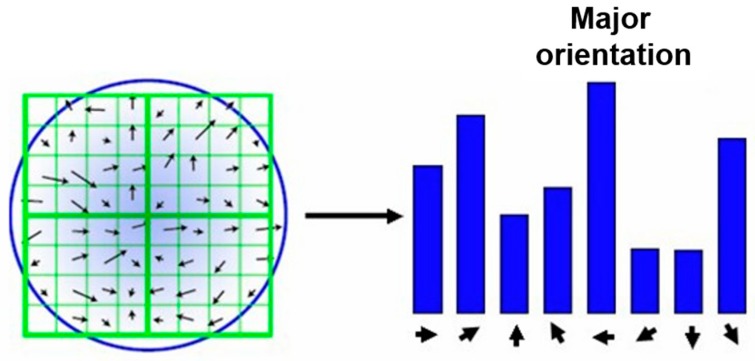
The orientation histogram of a feature point.

An orientation histogram is formed from the gradient orientations of the sample points within a region around the feature point. It has 36 bins covering the 360° range of orientations. Each bin covers 10 degrees. As shown in Figure 5, the peak in the histogram, indicating the dominant orientation of the local gradient image, is assigned as the orientation of the feature point.

**Figure 6:**
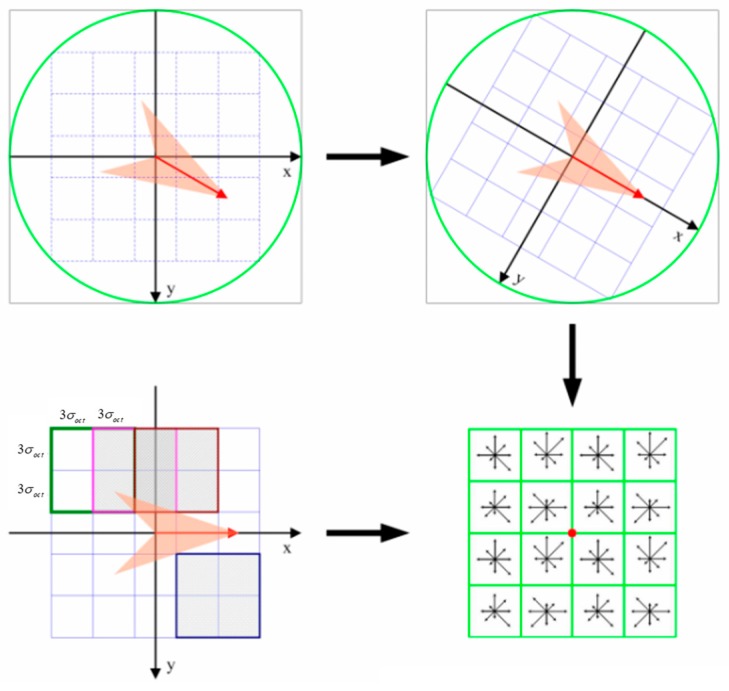
The feature descriptor of a feature point.

After determining the orientation of a feature point, the SIFT descriptor of the feature point is calculated using three steps, as illustrated in Figure 6.

First, the coordinates of the descriptor and the gradient orientations are rotated relative to the orientation of the feature point to achieve orientation invariance. Then, the orientation histograms over 4×4 sample regions centered with the feature point are created. There are eight directions for each orientation histogram, in which the length of each arrow represents the size of that histogram entry. Therefore, Lowe suggested using a 4 × 4 × 8 = 128 element feature vector for each feature point. Finally, the feature vector is normalized to unit length to reduce the effects of illumination change.

#### 2.4.3. Feature Matching

The BBF algorithm, based on a multidimensional space segmentation tree (K-D tree) [36], was adopted for feature matching in this work. The BBF algorithm can quickly find the nearest neighbor point and the second-closest neighbor point of a feature point to be matched. However, due to image noise or other reasons, the distance of the second nearest neighbor (*D_SN_*) may be very close to that of the nearest neighbor (*D_N_*). To reduce the impact of noise, the proposed method calculates the distance ratio, denoted as *R*, of *D_N_* to *D_SN_ (R = D_N_/D_SN_*). The nearest neighbor point is accepted as the match point of the feature point if *R* is higher than a default value of 0.8.

It is worth noting that the image coordinates of the matched points within each image patch need to be converted to the coordinates of the entire image scene. 

### 2.5. Removing the Outliers

The random sample consensus (RANSAC) algorithm is commonly used to remove incorrect matches obtained using the SIFT algorithm [37]. The RANSAC algorithm was proposed by Fischler and Bolles in 1981 [38]. It is an outlier detection method [39] that iteratively derives the parameters of a mathematical model from a set of observed data that contains outliers. However, because of the randomness of RANSAC, the effect of removing incorrect matches depends on the selection of sample points. To achieve robust performances, the RANSAC algorithm was combined with the homography matrix to remove outliers in this work. The homography matrix is a transformation matrix that maps the points in one image to the corresponding points in the other image. It can be represented using Equation (11).

(11) $$H=\left[\begin{array}{ccc} h_{00} & h_{01} & h_{02}\\ h_{10} & h_{11} & h_{12}\\ h_{20} & h_{21} & h_{22} \end{array}\right],$$

The matched tie points are denoted as {(*x_w_, y_w_*), *(x, y)*}, where (*x_w_, y_w_*) are the coordinates of the feature point in the input image to be registered, and *(x, y)* are the coordinates of the corresponding feature point in the reference image. The incorrectly matched tie points were excluded using the following steps.

First, a homography matrix *H* between the input image and the reference image was initially estimated by the RANSAC algorithm using all the matched tie points.

Then, the transformed coordinates of each feature point to be registered were estimated using the initial homography matrix *H*, according to Equation (12).

(12) $$\left[\begin{array}{c} X\\ Y\\ 1 \end{array}\right]=H\left[\begin{array}{c} x_{w}\\ y_{w}\\ 1 \end{array}\right]=\left[\begin{array}{ccc} h_{00} & h_{01} & h_{02}\\ h_{10} & h_{11} & h_{12}\\ h_{20} & h_{21} & h_{22} \end{array}\right]\left[\begin{array}{c} x_{w}\\ y_{w}\\ 1 \end{array}\right],$$

where *(x_w_, y_w_)* is the coordinates of the feature points of the input image and *(X, Y)* is the transformed coordinates of *(x_w_, y_w_)*.

After that, the residual error and root-mean-square error *(RMSE)* of the *i*th tie point were obtained using Equations (13) and (14), respectively,

(13) $$\begin{array}{c} \Delta x_{i}=x_{i}-X_{i}\\ \Delta y_{i}=y_{i}-Y_{i} \end{array},$$

(14) $$RMSE_{i}=\sqrt{\Delta x_{i}{}^{2}+\Delta y_{i}{}^{2}},$$

where Δ*x_i_* and Δ*y_i_* are the residual errors of the *i*th feature point and *x_i_* and *y_i_* are the coordinates of the *i*th feature point in the reference image. Tie point pairs that have *(*Δ*x_i_,* Δ*y_i_)* and *RMSE_i_* values higher than an initial threshold, which is denoted as *T_R_*, are considered outliers and removed.

Finally, *H* and *RMSE_i_* were estimated iteratively based on the remaining tie points and the outliers were removed until either the Δ*x_i_,* Δ*y_i_* and *RMSE_i_* values were lower than the threshold *T_R_* or the number of iterations was higher than a set value denoted as *N_iter_*.

### 2.6. Even Spatial Distribution of Matched Tie Points

The uneven spatial distribution of the matched tie points may lead to local geometric distortions of the rectified images. In addition, redundant tie points covering some local regions may increase the number of computations and therefore reduce the efficiency of image processing. To solve this problem, a greedy algorithm [40] was used in this work to remove matched tie points with high density.

Given that a number of *N*_tp_ tie point pairs were obtained after the elimination of the outliers, the following steps were performed to achieve the even distribution of the tie points.

Step 1: The perspective transform model between the input image and the reference image was estimated using all the matched tie points. The *RMSE* value of each tie point pair was calculated, and all the tie point pairs were then sorted in ascending order according to the *RMSE* values.
Step 2: The sorted tie point pairs were numbered according to the *RMSE* values. The first item of the sorted tie point pairs, which is numbered as 1 (i.e., *i* = 1), was selected as the first node.
Step 3: The distances between tie point *i* and the other tie points *j* (*j* = *i*+1, …, *N*_tp_) were calculated using the Euclidean distance. If the distance of a tie point *j* was less than a threshold (*T_L_*), this tie point was considered to be too close to the tie point *i*. Then, this tie point was removed, and let *i = i+1*.
Step 4: Step 3 was repeated until *i* = *N*_tp_.

**Figure 7:**
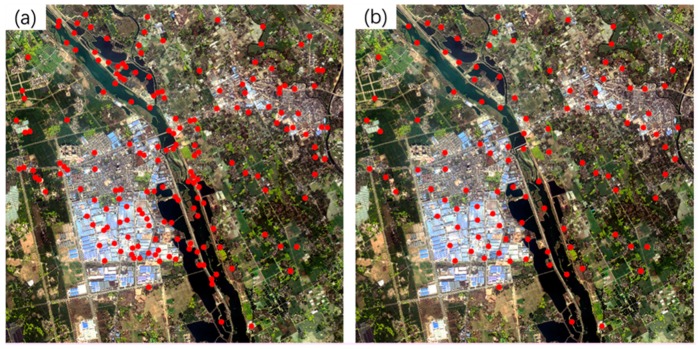
Comparison of the spatial distribution of the initial matched tie points (**a**) and the remaining tie points after removing some tie points with high density (**b**).

To test the employed approach for the even distribution of tie points, an experiment was performed using an 800 × 800 RS image (experimental results are shown in Figure 7). As shown in Figure 7a, 181 matched tie points (dots in red) with a model error of 0.93 pixels were obtained after removing outliers using the combination of RANSAC and the homography matrix. After removing high-density tie point pairs using a threshold (*T_L_*) of 30 pixels, 108 tie points with a model error of 0.75 pixels remained, as shown in Figure 7b. The remaining tie points were more evenly distributed and offered higher accuracy than the initial tie points.

### 2.7. Image Transformation and Resampling

In this paper, the polynomial rectification model was used to warp the input image. The coefficients of the polynomial model were solved using the matched tie points. The number of matched tie points should meet the requirement in Equation (15),

(15) $$N_{cp}\geq \left(N_{d}+1\right)\left(N_{d}+2\right)/2,$$

where *N_cp_* is the number of tie point pairs and *N_d_* is the degree of the polynomial model. The value of *N_d_* is determined according to *N_cp_* and the terrain relief of the images. When *N_d_* is 2 or 3, the polynomial model is applicable for topographic relief areas, such as mountain areas. After the coefficients were obtained, the input image was transformed and resampled to align it with the reference image.

## 3. Experiments and Results

In this section, three groups of postearthquake RS images were used to compare the proposed method with two other methods, including the classical SIFT (referred to as SIFT henceforth) and the SIFT method using the same patch matching approach (referred to as Patch-SIFT) used by the proposed method. The details of the three datasets (such as the satellite, resolution, size, acquired date, and disaster information) are introduced in Section 3.1. The evaluation criteria are presented in Section 3.2. The comparison results are displayed in Section 3.3.

The experiments were performed on a computer with an Intel Core i7-4770 CPU 3.40-GHz processor and 14.0 GB of physical memory, using Visual Studio 2013 (C#) as the programming environment.

### 3.1. Datasets

**Figure 8:**
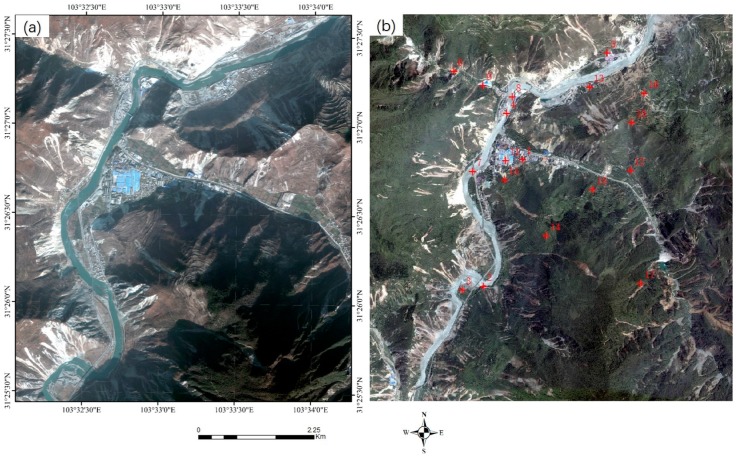
The Wenchuan earthquake dataset. The left (**a**) is the post-earthquake image (the input image), whereas the right (**b**) is the reference image in the pre-earthquake database. The points in red are the verification points used for calculating the accuracy of the rectified image.

**Figure 9:**
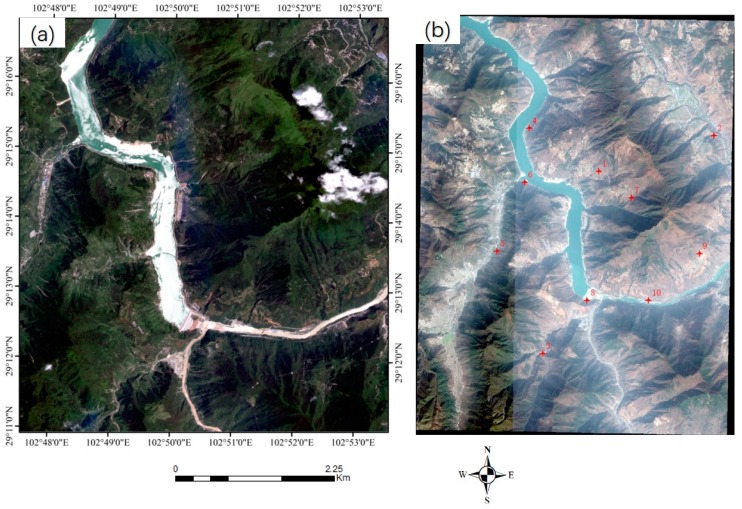
The Yaan earthquake dataset. The left (**a**) is the post-earthquake image (the input image), and the right (**b**) is the reference image in the pre-earthquake database. The points in red are the verification points used for calculating the accuracy of the rectified image.

**Figure 10:**
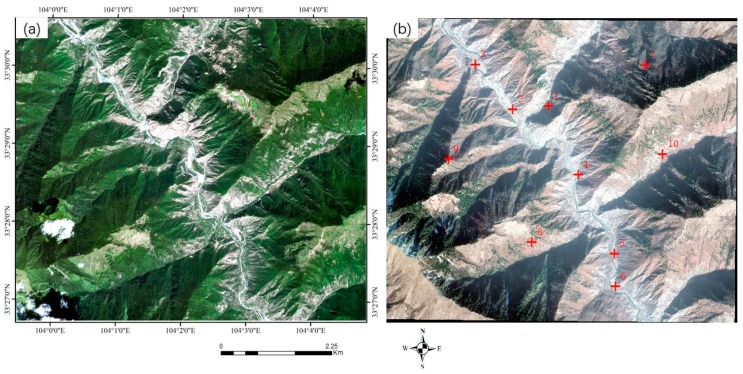
The Jiuzhaigou earthquake dataset. The left (**a**) is the post-earthquake image (the input image), and the right (**b**) is the reference image in the pre-earthquake database. The points in red are the verification points used for calculating the accuracy of the rectified image.

Three sets of high-resolution RS images after earthquakes were used in the experiment. All the images contain different secondary disasters caused by severe earthquakes. The first image is the QuickBird multispectral image (2.4 m) of the Wenchuan area that suffered from the Wenchuan earthquake (magnitude 8.2) on 12 May 2018. The image, with a size of 2427 × 2569 pixels, was acquired on 26 December 2008. The elevation of the image ranges from 1346 to 2960 m, as shown in Figure 8a. A large number of landslides can be seen in the image, as well as the reference image employed (Figure 8b). The second post-earthquake image is a GaoFen-1 (GF-1) multispectral image (8 m) covering Baoxing Town, which experienced the Yaan earthquake (magnitude 7.0) on 20 April 2013. The GF-1 image, recorded on 23 July 2013, has a size of 1218 × 1363 pixels. Both landslides and river expansion caused by the earthquake can be observed in the image, as shown in Figure 9a. Its elevation ranges from 711 to 2406 m. The third image is a GaoFen-2 (GF-2) image of the Jiuzhaigou area after the 7.0-magnitude Jiuzhaigou earthquake on 8 August 2017. The image was acquired on 9 August 2017, and has a size of 2096 × 1789 pixels. Its elevation, from 1892 to 3760 m, has a larger span. Landslides caused by the earthquake can be seen in the image, as shown in Figure 10a.

The details of the three post-disaster images and the corresponding reference images obtained from the pre-earthquake image database are presented in Table 2. The reference images are also shown on the right of Figure 8, Figure 9 and Figure 10.

According to the characteristics of the three datasets, the parameter settings of the proposed method are presented in Table 3.

### 3.2. Evaluation Criteria

The performance of the proposed method was evaluated using four criteria, including the number of matched tie points (*N_cp_*), the used time for image registration (*T*), the accuracy of the transformation model obtained using the final tie points (*RMSE_M_*), and the geometric accuracy of the registered image using some verification points (*RMSE_T_*).

The *RMSE_M_* and *RMSE_T_* are computed using Equation (16),

(16) $$RMSE=\sqrt{\frac{1}{N}\sum_{i=1}^{N}\left({\left(x_{i}-X_{i}\right)}^{2}+{\left(y_{i}-Y_{i}\right)}^{2}\right)},$$

where *(x_i_, y_i_)* are the projected coordinates of the *i*th point on the pre-earthquake image, *(X_i_, Y_i_)* are the projected coordinates of the *i*th point after transformation, and *N* is the number of points. The *RMSE_M_* is calculated using all matched tie points obtained by the three methods. The *RMSE_T_* can be calculated using some manually selected verification points. In this case, *(X_i_, Y_i_)* are the projected coordinates of the validation points in the reference image, whereas *(x_i_, y_i_)* are the coordinates of the corresponding points in the registered image.

To verify the geometric accuracies of the registered images, 18, 10, and 10 verification points were selected for the three datasets, respectively, using ENVI software. As shown in the reference images in Figure 8, Figure 9 and Figure 10, these verification points (shown in red), which are evenly distributed, were used to calculate the *RMSE_T_* of the three methods.

### 3.3. Experimental Results

Table 4 shows the statistics of the matched tie points *N*, running time *T*, model error *RMSE_M_*, and verification error *RMSE_T_* of the three datasets using the three algorithms. Both the quadratic (*N*_d_ = 2) and cubic (*N*_d_ = 3) polynomial models were considered in the experiments.

#### 3.3.1. Experiment 1 Using the Wenchuan Dataset

In the experiment using the first image pair, the proposed method obtained 52 matched tie points, which is slightly more than that of the Patch-SIFT method (*N_cp_* = 45) but significantly more than that of the SIFT method (*N_cp_* = 21). The proposed method yielded very similar performances when using the quadratic and cubic polynomial models. Using the cubic polynomial model, the proposed method yielded an *RMSE_T_* value of 1.40 pixels, which is significantly lower than those of the Patch-SIFT (*RMSE_T_* = 2.01 pixels) and SIFT (*RMSE_T_* = 9.18 pixels) methods. Although the SIFT method provided the lowest *RMSE_T_* value (0.81 pixels), the *RMSE_T_* value of 9.18 pixels is extremely high. Using the cubic polynomial model, the running time of the proposed method was 2.20 s, which is only 6.47% of the SIFT (33.98 s) method and 44.27% of the Patch-SIFT (4.97 s) method. Similarly, the proposed method using the quadratic polynomial model gave a lower *RMSE_T_* value and a shorter running time than the other two methods.

**Figure 11:**
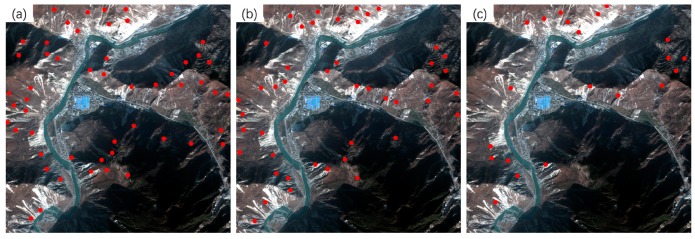
Matched tie points obtained from the Wenchuan dataset. (**a**) The proposed method; (**b**) Patch- scale-invariant feature transform (SIFT); and (**c**) SIFT.

Figure 11 shows the spatial distribution of matched tie points (dots in red) of the first image pair. It can be seen that the distribution of matched tie points obtained by the proposed method, as shown in Figure 11a, is the most even. As can be seen from Figure 11c, the 21 matched tie points obtained by the SIFT method are unevenly distributed, which contributes to the extremely high validation error it yielded. In contrast, the proposed method can still extract correct tie points in the areas with large topographic relief, such as the top-left corner of images shown in Figure 11.

**Figure 12:**
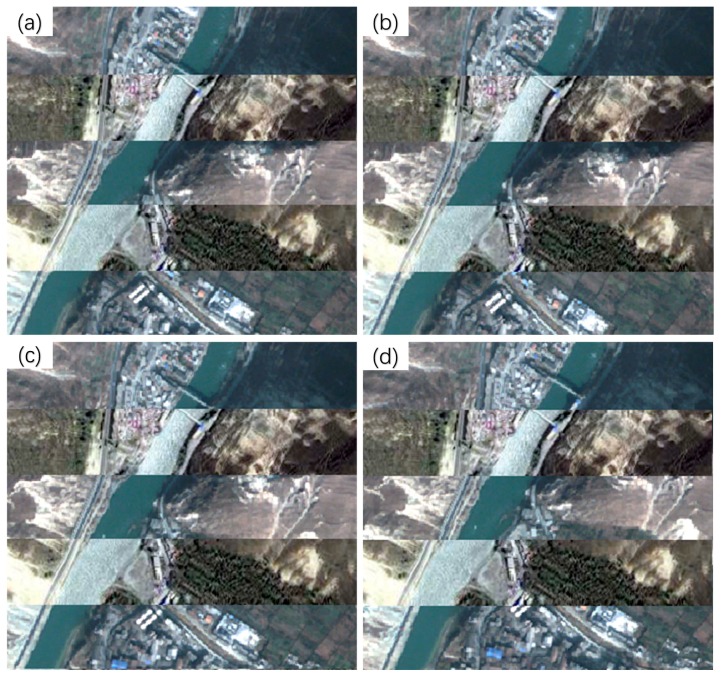
Registered images of the Wenchuan dataset. (**a**) The original image; (**b**) registered image using the proposed method; (**c**) registered image using Patch-SIFT; and (**d**) registered image using SIFT.

The registration results of the Wenchuan dataset using the cubic polynormal model are shown in Figure 12. It can be seen that only the registered image obtained using the proposed method (Figure 12b) can effectively rectify the geometric position deviations of the objects on the original image (Figure 12a). In contrast, the registration results of the Patch-SIFT (Figure 12c) and the SIFT (Figure 12d) methods show more significant displacements than the original image. This is consistent with the higher *RMSE_T_* values offered by the two methods. The main reason is that the number of matched tie points extracted by the two methods was small, and the spatial distribution of the tie points was uneven.

#### 3.3.2. Experiment 2 Using the Yaan Dataset

**Figure 13:**
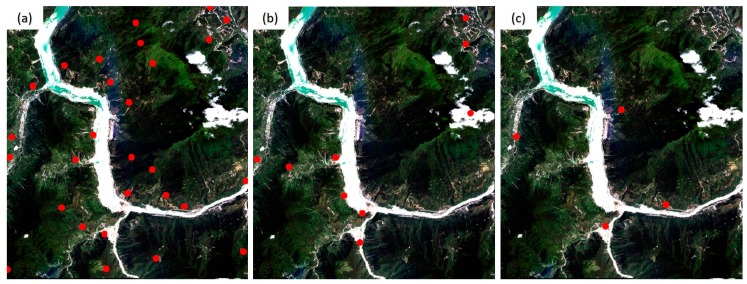
Matched tie points of the GF-1 image. (**a**) The proposed method, (**b**) Patch-SIFT, and (**c**) SIFT.

For the second image pair, the proposed method also obtained 29 matched tie points, which is significantly larger than those of the Patch-SIFT (*N_cp_* = 9) and SIFT (*N_cp_* = 4) methods. Since the numbers of tie points extracted by the Patch-SIFT method were less than the minimum number required for the cubic polynomial model, only the quadratic polynomial model was used for the Patch-SIFT method. For a similar reason, only the linear polynomial model was used for the SIFT method. Using the cubic polynomial model, the proposed method yielded a running time of 1.56 s, an *RMSE_M_* value of 1.81 pixels, and an *RMSE_T_* value of 1.75 pixels. The running time is only 34.29% of the SIFT (4.55 s) and 48.30% of the Patch-SIFT (3.23 s). The *RMSE_M_* and *RMSE_T_* values are also significantly lower than those of the other two methods. Similarly to the first image pair, the running time, *RMSE_M_*, and *RMSE_T_* of the proposed method obtained using the quadratic polynomial model were very close to those obtained using the cubic polynomial model. Similarly, the distribution of the matched tie points extracted by the proposed method (Figure 13a) is also more even than those of the Patch-SIFT (Figure 13b) and the SIFT (Figure 13c) methods.

**Figure 14:**
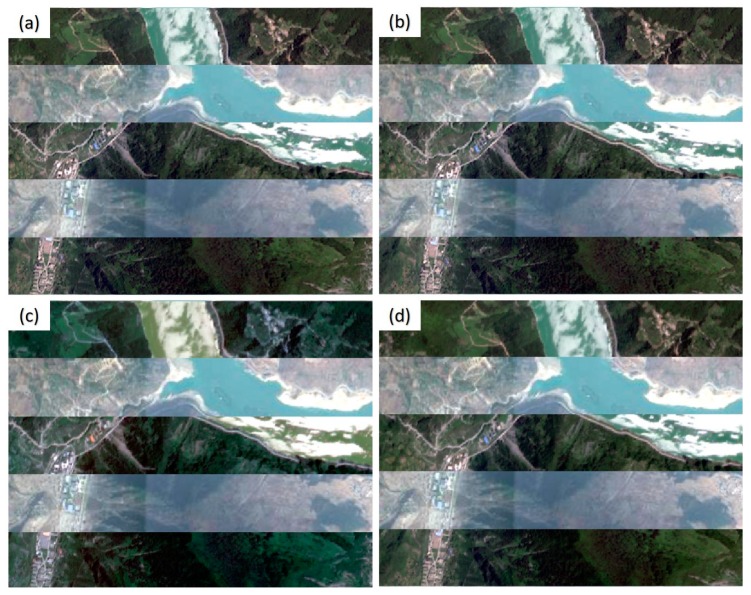
Registration results of the Yaan dataset. (**a**) The original image; (**b**) registered image of the proposed method; (**c**) registered image of Patch-SIFT; and (**d**) registered image of SIFT.

The registration results of the Yaan dataset are shown in Figure 14. Although there was a slight displacement for the road along the river in the registered image produced by the proposed method, the proposed method yielded better performance than the other two methods. In contrast, the registration results of Patch-SIFT (Figure 14c) and SIFT (Figure 14d) show even more noticeable displacements for the roads and constructions than the original image (shown in Figure 14a).

#### 3.3.3. Experiment 3 Using the Jiuzhaigou Dataset

**Figure 15:**
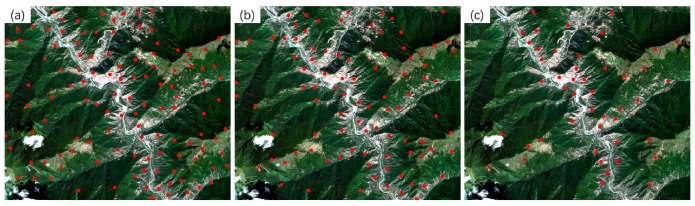
Matched tie points of the GF-2 image. (**a**) The proposed method; (**b**) Patch-SIFT; and (**c**) SIFT.

The third image pair shows significant differences in temporal and hue. In the experiment, the proposed method also provided the largest number of tie points (*N_cp_* = 118), whereas the Patch-SIFT and SIFT methods yielded 94 and 46 tie points, respectively. The proposed method has a significant advantage in the running time over the other two methods. The running times of the proposed method were 2.05 and 1.93 s for the cubic and quadratic polynomial models, respectively. The former is only 7.36% of the SIFT method (27.85 s) and 32.23% of the Patch-SIFT method (6.36 s) using the same model. The proposed method provided *RMSE_M_* values of 1.37 and 1.39 pixels, respectively, which are higher than those of the Patch-SIFT (*RMSE_M_* = 1.27 pixels and *RMSE_M_* = 1.32 pixels, respectively) and SIFT (*RMSE_M_* = 0.72 pixels and *RMSE_M_* = 0.76 pixels, respectively) methods. Although the SIFT method yielded the lowest model error, the validation error (*RMSE_T_* = 2.48 and *RMSE_T_* = 2.51 pixels for the cubic and quadratic polynomial models, respectively) was extremely high. The main reason is that the number of matched tie points was small, and the spatial distribution of tie points was uneven. The spatial distribution of matched tie points is shown in Figure 15. It can be seen that the spatial distribution of the matched tie points (dots in red) extracted using the proposed method (as shown in Figure 15a) was the most even. In contrast, the matched tie points obtained by the SIFT are mainly located in the image patches with significant features (such as roads and houses). However, in the image patches with complex terrain, the number of matched tie points extracted by the SIFT method was very limited compared with the two methods using the image partitioning strategy.

**Figure 16:**
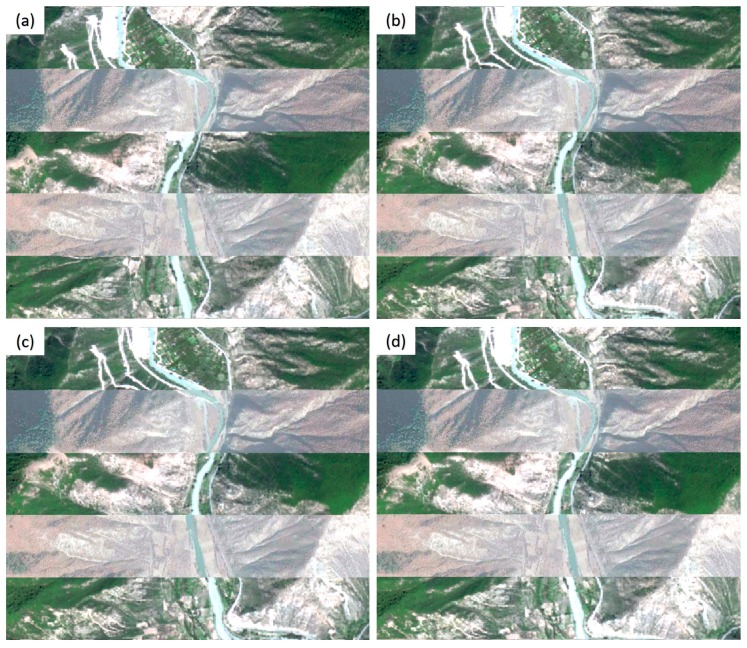
Registered images for the Jiuzhaigou dataset. (**a**) The original image; (**b**) the registered image of the proposed method; (**c**) the registered image of the Patch-SIFT method; and (**d**) the registered image of the SIFT method.

The registered images of the Jiuzhaigou dataset using the cubic polynormal model are shown in Figure 16. As shown in Figure 16b, the registered image of the proposed method still had slight displacements with the original image. In contrast, the other two methods had noticeable displacements for rivers and roads (Figure 16c,d). This is also consistent with the *RMSE_T_* values provided by the three methods. Consequently, compared with the other methods, the proposed method can achieve a larger number of tie points, which are more evenly distributed. This is helpful for improving the accuracy of image registration.

## 4. Discussions

The experimental results demonstrate the advantages of the proposed method over the SIFT and Patch-SIFT methods in terms of accuracy, running time, and the number and distribution of matched tie points for the three sets of pre- and post-earthquake images. All three image pairs covered mountainous areas affected by disasters (such as landslides and river expansion, triggered by severe earthquakes). Topographic relief in the images increased the difficulty of image registration. However, in this case, the proposed method still achieved better registration results.

Both the quadratic (*N*_d_ = 2) and cubic (*N*_d_ = 3) polynomial models were used in the experiments to generate registered images. The proposed method yielded very similar performances when using the two models. Using the quadratic polynomial model (*N*_d_ = 2), the proposed method also provided shorter running times and lower *RMSE_T_* values than the Patch-SIFT and SIFT methods using the same model. The proposed method using the cubic polynomial model yielded slightly longer running times and lower *RMSE_T_* values than the proposed method using the quadratic polynomial model. Consequently, we think that both the quadratic and cubic polynomial models can be selected for the images used in the experiments.

The proposed method can be also used for satellite images (such as GF-1 and GF-2 images) covering plains, according to the results of the other experiments. For example, a GF-1 multispectral image covering Chengdu City, Sichuan province, China, was also tested. The image was recorded on March 9, 2018 and had a relatively large size (5354 × 5354 pixels) and a spatial resolution of 8 m. A total of 175 matched tie points were extracted by the proposed method, with an *RMSE_T_* of 0.38 pixels. The Patch-SIFT method obtained 116 matched tie points with an *RMSE_T_* of 0.55 pixels, whereas the SIFT method provided 80 tie points with an *RMSE_T_* of 0.66 pixels. The proposed method obtained the registered image within 17.17 s, which is only 26.5% of the SIFT (64.77 s) and 36.7% of the Patch-SIFT (46.76 s). The experimental results also show that the registration results of the proposed method are better than those of the other two methods. This demonstrates that the proposed method also works for RS images covering urban areas and shows advantages in running time on larger RS images.

The GE images were used in this work to construct the pre-earthquake image database and then used as the reference for the co-registration of post-earthquake RS images. If higher quality high-resolution satellite remote sensing images were available in the study area, the images could be used as the reference for image registration. In this case, the proposed method is also applicable and is expected to yield better performance than the other two methods.

In general, the proposed method can provide geometrically accurate images for disaster information extraction, and is helpful for the assessment of disasters, such as collapsed buildings, damaged roads, and landslides. The proposed method also has potential applications. For instance, the registered images obtained by the proposed method can be integrated with big data, such as the Floating Car Data [41], which is used to observe historical traffic patterns, traffic facilities, and services, to the phenomenon after the earthquakes.

## 5. Conclusions

To improve the efficiency and accuracy of multisource remote sensing image registration in the process of earthquake disaster assessment, a new automatic registration method for multisource RS images was proposed in this paper. Based on the construction of a pre-earthquake database, the proposed method employed a combination of the Shi_Tomasi and SIFT methods to extract tie points using the image partitioning strategy based on geographic information constraints. Additionally, a greedy algorithm was introduced to eliminate redundant tie points and thus to achieve even spatial distribution of matched tie points. The accuracy and robustness of the proposed method were compared with the traditional SIFT method and the Patch-SIFT method using three pairs of high-resolution images covering regions affected by severe earthquakes. The experimental results show that the proposed method outperformed the other two methods in terms of the accuracy of the registered image, running time, and number and distribution of matched tie points. The larger number and more even distribution of the tie points extracted by the proposed method contribute to the improvement in accuracy. The experimental results indicate that the proposed method is more suitable than the Patch-SIFT and SIFT methods in the case of the registration of post-earthquake high-resolution RS images used for earthquake damage assessment. According to the experimental results of a test using a GF-1 image covering urban areas (with a size of 5354 × 5354 pixels), the proposed method can also be used for satellite images covering plains (including urban areas), and shows significant advantages in running time over the two comparison methods for large-size RS images.

## Figures and Tables

**Table 1:** The information in the coordinate files of the pre-earthquake remote sensing (RS) image database.

Field Name	Description
ID_Img	The ID of pre-earthquake images
Ref_Lon_Top	Top longitude of a pre-earthquake RS image
Ref_Lat_Left	Left latitude of a pre-earthquake RS image
Ref_Lon_Bottom	Bottom longitude of a pre-earthquake RS image
Ref_Lat_Right	Right latitude of a pre-earthquake RS image
Ref_X	Center x (in projected coordinates) of a pre-earthquake RS image
Ref_Y	Center y (in projected coordinates) of a pre-earthquake RS image
Ref_Pixelsize	The spatial resolution of a pre-earthquake RS image

**Table 2:** Details of the three image datasets used in the experiment.

No.	Satellite	Resolution (m)	Size (pixel)	Date	Earthquake	Disaster
1	QuickBird	2.4	2427 × 2569	2008/12/26	2008 Wenchuan earthquake	Landslides
	GE	2	2932 × 3108	2008/05/23		
2	GF-1	8	1218 × 1363	2013/07/23	2013 Yaan earthquake.	Landslides, river expansion
	GE	2	6568 × 8644	2010/02/08		
3	GF-2	4	2096 × 1789	2017/08/09	2017 Jiuzhaigou earthquake.	Landslides
	GE	2	4632 × 4002	2014/02/05		

**Table 3:** Parameter settings for the three sets of experimental data.

Parameters	Parameters Setting			Specification of Parameters
	1	2	3	
**n**	10 × 10	10 × 10	7 × 7	The number of image patches
N_max_	1500	1500	1500	The maximum number of corner points for each image patch
N_iter_	10	10	10	The number of iterations for eliminating mismatched tie points
T_R_/pixel	5	3	5	The residual threshold for eliminating mismatched tie points
T_L_/pixel	100	100	30	The distance threshold for removing high-density tie points

**Table 4:** Experimental results for three sets of images.

Image Pair	Method	*N_cp_* (pair)	*N_d_*	*T* (s)	*RMSE_M_* (pixel)	*RMSE_T_* (pixel)
1	The proposed method	52	3	2.20	1.65	1.40
			2	1.95	1.79	1.95
	Patch-SIFT	45	3	4.97	1.94	2.01
			2	4.91	2.07	2.27
	SIFT	21	3	33.98	0.81	9.18
			2	33.89	1.24	9.27
2	The proposed method	29	3	1.56	1.81	1.75
			2	1.48	1.93	1.95
	Patch-SIFT	9	2	3.23	2.39	2.76
	SIFT	4	1	4.55	2.69	5.10
3	The proposed method	118	3	2.05	1.37	1.24
			2	1.93	1.39	1.30
	Patch-SIFT	94	3	6.36	1.27	1.88
			2	6.31	1.32	1.92
	SIFT	46	3	27.85	0.72	2.48
			2	27.88	0.76	2.51
